# Electrophysiology for Clinicians. Editors: Miguel A Barrero Garcia, Paul Khairy, Laurent Macle, Stanely Nattel. Cardiotext Publishing, LLC

**Published:** 2012-01-31

**Authors:** Johnson Francis

**Affiliations:** 1 Senior Consultant Cardiologist, Baby Memorial Hospital, Calicut, Kerala, India; 2 Professor of Cardiology, KMCT Medical College, Calicut, Kerala, India

Cardiac electrophysiology is a rapidly expanding sub speciality of cardiology with ever increasing new information coming in every day, with quite a few journals devoted to it. Complexity of arrhythmias are sometimes puzzling for a busy cardiologist and so are electrophysiology studies. *Electrophysiology for Clinicians* is a concise book on basic concepts of cardiac electrophysiology for the busy clinician.

*Electrophysiology for Clinicians*is a 254 page book divided into four parts and 13 chapters. In addition to four editors, the book has five contributors, all engaged in active electrophysiology work and research. Unlike most multi authored text books, most of the chapters are either written or co-authored by the editors themselves. This naturally reduces the redundancy between the chapters and maintains a uniformity in the pattern. Initial chapter is on basic cardiac electrophysiology with brief description of the elctrical system of the heart, ion channels and their regulation as well the most important description of the cardiac action potential. Second chapter on antiarrhythmic drugs covers the basic clinical pharmacology of commonly used anti arrhythmic drugs.

Part two of the book has two chapters on disorders of impulse formation and conduction describing sinoatrial node disorders and atrioventricular conduction disorders. Third part is on syncope, sudden cardiac death and clinical arrhythmias. Each chapter starts of with general principles, goes on to describing mechanism and then the clinical history, physical examination and further evaluation in that order. Illustrative ECG tracings are given wherever needed. Indications for electrophysiologic study and devices are given as easy to read tables.

Fourth and final part of the book is fully devoted to devices. The chapter on pacemakers gives the various pacing modes and codes and also the principles of implantation and follow up. This chapter also includes a section on biventricular pacing in heart failure. Basic programming and trouble shooting are also included. The chapter on implantable defibrillators follows a similar pattern. The last chapter is on selected electrophysiology techniques which includes clinical procedures, electrophysiological procedures, non invasive testing as well as a note on antiocoagulation in electrophysiology. Overall this book aims to be an excellent tool in the practical management of electrophysiological disorders for the practicing cardiologist.

